# Top 100 most-cited articles on NLRP3 (2000–2024): a retrospective analysis

**DOI:** 10.3389/fimmu.2026.1745589

**Published:** 2026-03-17

**Authors:** Ke-qian Chen, Hai-bo Lei, Mei-ling Huang, Xiang Liu

**Affiliations:** 1 Department of Clinical Pharmacy, Xiangtan Central Hospital (The Affiliated Hospital of Hunan University), Xiangtan, China; 2 Otorhinolaryngology Head and Neck Surgery, Xiangtan Central Hospital (The Affiliated Hospital of Hunan University), Xiangtan, China

**Keywords:** inflammasome, NLRP3, nod-like receptor family pyrin domain containing 3, nod-like receptor protein 3, top 100 most-cited articles

## Abstract

**Objective:**

This study aims to identify and analyze the top 100 most-cited articles in the NLRP3 research field to uncover research trends, key contributors, and collaborative networks, thereby providing insights into the development and future directions of NLRP3 inflammasome studies.

**Methods:**

A comprehensive search was conducted using the Web of Science and PubMed from January 2000 to December 2024. Search terms included “NLRP3” and related nomenclature. Articles were screened and sorted by citation frequency, and the top 100 were selected for detailed analysis of citations, authors, countries, institutions, journals, funding sources, and research fields.

**Results:**

These articles covered 20 publishers, 218 funds, 19 research fields, 811 authors, 25 countries, 46 journals, and 224 institutions. Among these authors, Latz E published the largest number of articles and had the highest citation frequency and the strongest co-authorship ties. Among these countries, USA published the largest number of articles and had the highest citation frequency and the strongest co-authorship ties. Among these institutions, University of Massachusetts published the largest number of articles and had the highest citation frequency and the strongest co-authorship ties. Among these journals, Nature published the largest number of articles and had the highest citation frequency and the strongest co-authorship ties.

**Conclusion:**

This retrospective analysis highlights the growing interest and interdisciplinary nature of NLRP3 inflammasome research. The USA, leading institutions, and high-impact journals play central roles in shaping the field. Future research is expected to focus on translational applications, including the development of NLRP3 inhibitors and activators, and exploring NLRP3 functions in non-immune cells.

## Introduction

In the human immune defense network, innate immunity is our first line of defense (1, 2). In this line of defense, a number of protein complexes play a key role as “hazard sensors” (3). As an important protein complex, inflammasomes are divided into seven members: NLRP1, NLRP2, NLRP3, NLRP6, NLRP12, NLRC4, and AIM2 (4, 5). Among these inflammasomes, the most widely studied is the NLRP3 inflammasome (6). The NLRP3 inflammasome is composed of NLRP3, ASC, and Caspase-1 (7). Its activation includes NLRP3 inflammasome assembly, Caspase-1 activation, and cleavage and processing of Pro-IL-1β and Pro-IL-18. Finally, IL-1β and IL-18 are secreted into the extracellular space to trigger an inflammatory response (8). Pathogenic microorganisms, silica, cholesterol crystals, urate, adenosine triphosphate (ATP), and reactive oxygen species (ROS) often act as activators of NLRP3 inflammasome (9–11). Post-translational modifications play a key role in this process (12). Over the past two decades, more and more studies have begun to delve into the NLRP3 inflammasome. These studies have shown that the activation pathways of NLRP3 inflammasome mainly include intracellular ion flow, mitochondrial damage, Golgi disassembly, lysosomal cleavage, and endoplasmic reticulum stress (13–15). The NLRP3 inflammasome has become an attractive therapeutic target in many diseases (16). Many NLRP3 activators and NLRP3 inhibitors are also being developed by researchers (17–19).

The number of citations of an article is an important indicator for measuring academic influence and research value. On the one hand, the number of citations of an article can reflect its popularity. On the other hand, the number of citations can prove the contribution and influence of the article in a certain field. By conducting a comprehensive analysis of the top 100 most-cited articles, researchers can effectively understand the development history and future direction of a research field. This study aims to explore the top 100 most-cited articles in the NLRP3 field and identify the research trends in this field. This will provide valuable references for future research in this field.

## Methods

**Figure 1:**
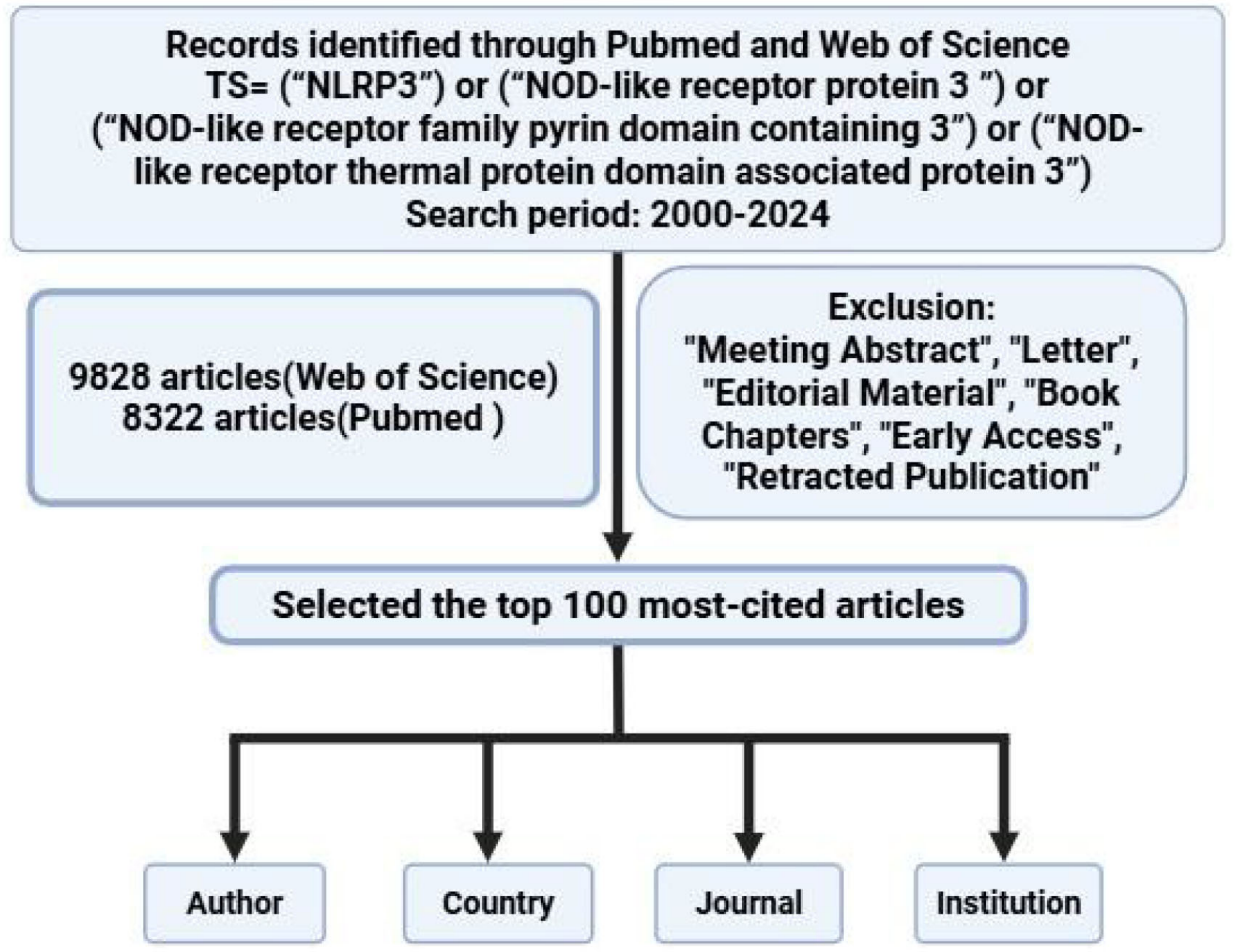
Flow chart of retrospective analysis.

A comprehensive search was conducted in the Web of Science Core Collection and PubMed databases on December 31, 2024. The exact search queries were as follows: Web of Science: TS = (“NLRP3” OR “NOD-like receptor protein 3” OR “NOD-like receptor family pyrin domain containing 3” OR “NOD-like receptor thermal protein domain associated protein 3”). PubMed: (“NLRP3”[Title/Abstract] OR “NOD-like receptor protein 3”[Title/Abstract] OR “NOD-like receptor family pyrin domain containing 3”[Title/Abstract] OR “NOD-like receptor thermal protein domain associated protein 3”[Title/Abstract]). The search was limited to articles and review articles published between January 1, 2000 and December 31, 2024. After retrieval, duplicates between the two databases were identified using EndNote X9 and manually verified, then removed. A total of 9828 articles were included (**Figure 1**). We have adhered to PRISMA guidelines. Two researchers independently reviewed these articles, sorted them by citation frequency from highest to lowest, selected the top 100 most-cited articles, and extracted the relevant data (citation frequency, author, country, institution, journal, year, keyword, research field, funding, publisher). Citation counts were extracted from the Web of Science Core Collection, and self-citations were not excluded as they are considered part of an article’s academic impact in bibliometric analysis.

## Result

**Figure 2:**
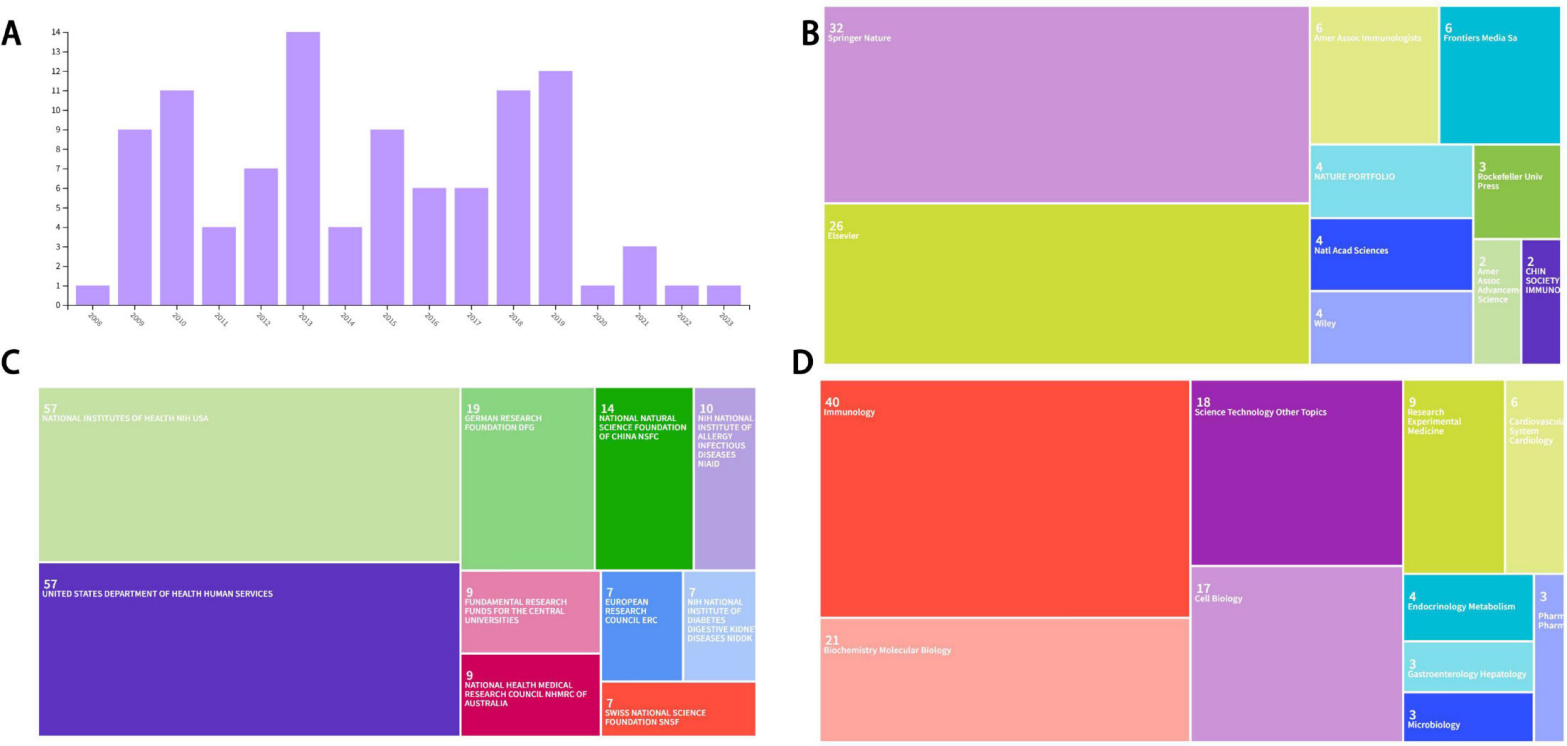
**(A)** Annual publication count for NLRP3 from 2015 to 2024. **(B)** Top 10 publishers of the 100 most-cited articles. **(C)** Top 10 funds of the 100 most-cited articles. **(D)** Top 10 research fields of the 100 most-cited articles.

The basic information of the 100 most-cited articles included 20 publishers, 218 funds, and 19 fields. As shown in **Figure 2A**, the 100 most-cited articles were published from 2008 to 2023. The year with the highest number of published articles was 2013 (14 articles), while the years with the lowest number of published articles were 2008, 2020, 2022, and 2023 (1 article each). As shown in **Table 1**, the article with the highest citation frequency (11856) was published in 2019, while the article with the lowest citation frequency (422) was published in 2020. Correlation analysis revealed a weak but significant negative correlation between publication year and total citations, indicating that older articles tend to accumulate more citations. However, when analyzing normalized citations per year (average), no significant correlation was observed. As shown in **Figure 2B**, the top 10 publishers were Springer Nature (32 articles), Elsevier (26 articles), American Association of Immunologists (6 articles), Frontiers Media SA (6 articles), Nature Portfolio (4 articles), National Academy of Sciences (4 articles), Wiley (4 articles), Rockefeller University Press (3 articles), American Association for the Advancement of Science (2 articles), Chinese Society for Immunology (2 articles). As shown in **Figure 2C**, the top 10 funds were National Institutes of Health (57 articles), United States Department of Health and Human Services (57 articles), German Research Foundation (19 articles), National Natural Science Foundation of China (14 articles), National Institute of Allergy and Infectious Diseases (10 articles), Fundamental Research Funds for the Central Universities (9 articles), National Health and Medical Research Council (9 articles), European Research Council (7 articles), National Institute of Diabetes and Digestive and Kidney Diseases (7 articles), Swiss National Science Foundation (7 articles). As shown in **Figure 2D**, the top 10 research fields were Immunology (40 articles), Biochemistry Molecular Biology (21 articles), Science Technology Other Topics (18 articles), Cell Biology (17 articles), Research Experimental Medicine (9 articles), Cardiovascular System Cardiology (6 articles), Endocrinology Metabolism (4 articles), Gastroenterology Hepatology (3 articles), Microbiology (3 articles), Pharmacology Pharmacy (3 articles).

**Table 1:** The information of top 100 cited articles in NLRP3.

Rank	First author	Year	Journal	Title	Citation
1	Zhou, RB	2011	Nature	A role for mitochondria in NLRP3 inflammasome activation	4406
2	Swanson, KV	2019	Nature Reviews Immunology	The NLRP3 inflammasome: Molecular activation and regulation to therapeutics	3293
3	Duewell, P	2010	Nature	NLRP3 inflammasomes are required for atherogenesis and activated by cholesterol crystals	3102
4	Kelley, N	2019	International Journal of Molecular Sciences	The NLRP3 inflammasome: An overview of mechanisms of activation and regulation	2486
5	Bauernfeind, FG	2009	Journal of Immunology	Cutting edge: NF-κB activating pattern recognition and cytokine receptors license NLRP3 inflammasome activation by regulating NLRP3 expression	2400
6	He, Y	2016	Trends in Biochemical Sciences	Mechanism and regulation of NLRP3 inflammasome activation	2165
7	Heneka, MT	2013	Nature	NLRP3 is activated in Alzheimer’s disease and contributes to pathology in APP/PS1 mice	2158
8	Coll, RC	2015	Nature Medicine	A small-molecule inhibitor of the NLRP3 inflammasome for the treatment of inflammatory diseases	2119
9	Vandanmagsar, B	2011	Nature Medicine	The NLRP3 inflammasome instigates obesity-induced inflammation and insulin resistance	2118
10	Shimada, K	2012	Immunity	Oxidized mitochondrial DNA activates the NLRP3 inflammasome during apoptosis	1706
11	Muñoz-Planillo, R	2013	Immunity	K^+^ efflux is the common trigger of NLRP3 inflammasome activation by bacterial toxins and particulate matter	1670
12	Mangan, MSJ	2018	Nature Reviews Drug Discovery	Targeting the NLRP3 inflammasome in inflammatory diseases	1597
13	Ghiringhelli, F	2009	Nature Medicine	Activation of the NLRP3 inflammasome in dendritic cells induces IL-1β-dependent adaptive immunity against tumors	1576
14	Youm, YH	2015	Nature Medicine	The ketone metabolite β-hydroxybutyrate blocks NLRP3 inflammasome-mediated inflammatory disease	1546
15	Tschopp, J	2010	Nature Reviews Immunology	NLRP3 inflammasome activation: The convergence of multiple signaling pathways on ROS production?	1506
16	Wen, HT	2011	Nature Immunology	Fatty acid-induced NLRP3-ASC inflammasome activation interferes with insulin signaling	1443
17	Masters, SL	2010	Nature Immunology	Activation of the NLRP3 inflammasome by islet amyloid polypeptide provides a mechanism for enhanced IL-1β in type 2 diabetes	1064
18	Jo, EK	2016	Cellular & Molecular Immunology	Molecular mechanisms regulating NLRP3 inflammasome activation	1061
19	Yang, Y	2019	Cell Death & Disease	Recent advances in the mechanisms of NLRP3 inflammasome activation and its inhibitors	987
20	Ising, C	2019	Nature	NLRP3 inflammasome activation drives tau pathology	981
21	He, Y	2016	Nature	NEK7 is an essential mediator of NLRP3 activation downstream of potassium efflux	963
22	Huang, Y	2021	Cellular & Molecular Immunology	NLRP3 inflammasome activation and cell death	961
23	Schroder, K	2010	Science	The NLRP3 inflammasome: A sensor for metabolic danger?	942
24	Allen, IC	2009	Immunity	The NLRP3 inflammasome mediates *in vivo* innate immunity to influenza a virus through recognition of viral rna	926
25	Zhong, ZY	2018	Nature	New mitochondrial DNA synthesis enables NLRP3 inflammasome activation	861
26	Lee, GS	2012	Nature	The calcium-sensing receptor regulates the NLRP3 inflammasome through Ca^2+^ and camp	840
27	Mridha, AR	2017	Journal of Hepatology	NLRP3 inflammasome blockade reduces liver inflammation and fibrosis in experimental NASH in mice	839
28	Yan, YQ	2015	Cell	Dopamine controls systemic inflammation through inhibition of NLRP3 inflammasome	835
29	Zaki, MH	2010	Immunity	The NLRP3 inflammasome protects against loss of epithelial integrity and mortality during experimental colitis	829
30	Rajamäki, K	2010	Plos One	Cholesterol crystals activate the NLRP3 inflammasome in human macrophages: A novel link between cholesterol metabolism and inflammation	794
31	Wree, A	2014	Hepatology	NLRP3 inflammasome activation results in hepatocyte pyroptosis, liver inflammation, and fibrosis in mice	777
32	Bauer, C	2010	Gut	Colitis induced in mice with dextran sulfate sodium (DSS) is mediated by the NLRP3 inflammasome	764
33	Sharma, BR	2021	Nature Immunology	NLRP3 inflammasome in cancer and metabolic diseases	760
34	Gross, O	2009	Nature	SYK kinase signaling couples to the NLRP3 inflammasome for anti-fungal host defense	756
35	Christ, A	2018	Cell	Western diet triggers NLRP3-dependent innate immune reprogramming	745
36	Sheedy, FJ	2013	Nature Immunology	CD36 coordinates NLRP3 inflammasome activation by facilitating intracellular nucleation of soluble ligands into particulate ligands in sterile inflammation	740
37	Iyer, SS	2013	Immunity	Mitochondrial cardiolipin is required for NLRP3 inflammasome activation	729
38	Abais, JM	2015	Antioxidants & Redox Signaling	Redox regulation of NLRP3 inflammasomes: ROS as trigger or effector?	728
39	Murakami, T	2012	Proceedings of The National Academy of Sciences of The United States of America	Critical role for calcium mobilization in activation of the NLRP3 inflammasome	726
40	Shao, BZ	2015	Frontiers in Pharmacology	NLRP3 inflammasome and its inhibitors: A review	708
41	Coll, RC	2019	Nature Chemical Biology	MCC950 directly targets the NLRP3 ATP- hydrolysis motif for inflammasome inhibition	704
42	Lerner, AG	2012	Cell Metabolism	IRE1α induces thioredoxin-interacting protein to activate the NLRP3 inflammasome and promote programmed cell death under irremediable ER stress	700
43	Elliott, EI	2015	Immunological Reviews	Initiation and perpetuation of NLRP3 inflammasome activation and assembly	689
44	Misawa, T	2013	Nature Immunology	Microtubule-driven spatial arrangement of mitochondria promotes activation of the NLRP3 inflammasome	688
45	Allen, IC	2010	Journal of Experimental Medicine	The NLRP3 inflammasome functions as a negative regulator of tumorigenesis during colitis-associated cancer	681
46	Baroja-Mazo, A	2014	Nature Immunology	The NLRP3 inflammasome is released as a particulate danger signal that amplifies the inflammatory response	647
47	Grebe, A	2018	Circulation Research	NLRP3 inflammasome and the IL-1 pathway in atherosclerosis	637
48	Afonina, IS	2017	Nature Immunology	Limiting inflammation-the negative regulation of NF-κB and the NLRP3 inflammasome	629
49	Bruchard, M	2013	Nature Medicine	Chemotherapy-triggered Cathepsin B release in myeloid-derived suppressor cells activates the NLRP3 inflammasome and promotes tumor growth	625
50	Juliana, C	2012	Journal of Biological Chemistry	Non-transcriptional priming and deubiquitination regulate NLRP3 inflammasome activation	625
51	Lee, HM	2013	Diabetes	Upregulated NLRP3 inflammasome activation in patients with type 2 diabetes	621
52	Thomas, PG	2009	Immunity	The intracellular sensor NLRP3 mediates key innate and healing responses to influenza a virus via the regulation of caspase-1	620
53	He, HB	2018	Nature Communications	Oridonin is a covalent NLRP3 inhibitor with strong anti-inflammasome activity	615
54	Abderrazak, A	2015	Redox Biology	NLRP3 inflammasome: From a danger signal sensor to a regulatory node of oxidative stress and inflammatory diseases	594
55	Yan, YQ	2013	Immunity	Omega-3 fatty acids prevent inflammation and metabolic disorder through inhibition of NLRP3 inflammasome activation	593
56	Kuriakose, T	2016	Science Immunology	ZBP1/DAI is an innate sensor of influenza virus triggering the NLRP3 inflammasome and programmed cell death pathways	591
57	Fu, JN	2023	Annual Review of Immunology	Structural mechanisms of NLRP3 inflammasome assembly and activation	585
58	Rathinam, VAK	2012	Cell	TRIF licenses caspase-11-dependent NLRP3 inflammasome activation by gram-negative bacteria	585
59	Jiang, H	2017	Journal of Experimental Medicine	Identification of a selective and direct NLRP3 inhibitor to treat inflammatory disorders	572
60	Iyer, SS	2009	Proceedings of The National Academy of Sciences of The United States of America	Necrotic cells trigger a sterile inflammatory response through the NLRP3 inflammasome	570
61	Sharif, H	2019	Nature	Structural mechanism for NEK7-licensed activation of NLRP3 inflammasome	567
62	Guo, CS	2016	Immunity	Bile acids control inflammation and metabolic disorder through inhibition of NLRP3 inflammasome	564
63	Py, BF	2013	Molecular Cell	Deubiquitination of NLRP3 by BRCC3 critically regulates inflammasome activity	560
64	Subramanian, N	2013	Cell	The adaptor mavs promotes NLRP3 mitochondrial localization and inflammasome activation	553
65	Li, HF	2008	Journal of Immunology	Cutting edge: Inflammasome activation by alum and alum’s adjuvant effect are mediated by NLRP3	540
66	Zahid, A	2019	Frontiers in Immunology	Pharmacological inhibitors of the NLRP3 inflammasome	538
67	Paik, S	2021	Cellular & Molecular Immunology	An update on the regulatory mechanisms of NLRP3 inflammasome activation	533
68	Lawlor, KE	2015	Nature Communications	RIPK3 promotes cell death and NLRP3 inflammasome activation in the absence of mlkl	532
69	Kim, HY	2014	Nature Medicine	Interleukin-17-producing innate lymphoid cells and the NLRP3 inflammasome facilitate obesity-associated airway hyperreactivity	531
70	Bauernfeind, F	2011	Journal of Immunology	Cutting edge: Reactive oxygen species inhibitors block priming, but not activation, of the NLRP3 inflammasome	526
71	Youm, YH	2013	Cell Metabolism	Canonical NLRP3 inflammasome links systemic low-grade inflammation to functional decline in aging	519
72	Zhen, Y	2019	Frontiers in Immunology	NLRP3 inflammasome and inflammatory bowel disease	515
73	Toldo, S	2018	Nature Reviews Cardiology	The NLRP3 inflammasome in acute myocardial infarction	498
74	Chen, JQ	2018	Nature	PTDINS4P on dispersed trans-golgi network mediates NLRP3 inflammasome activation	497
75	Sano, S	2018	Journal of The American College of Cardiology	TET2-mediated clonal hematopoiesis accelerates heart failure through a mechanism involving the IL-1β/NLRP3 inflammasome	496
76	Tarallo, V	2012	Cell	DICER1 loss and alu rna induce age-related macular degeneration via the NLRP3 inflammasome and myd88	494
77	Franchi, L	2009	Journal of Immunology	Cutting edge: TNF-α mediates sensitization to atp and silica via the NLRP3 inflammasome in the absence of microbial stimulation	490
78	Lin, QS	2019	Redox Biology	Pink1-parkin pathway of mitophagy protects against contrast-induced acute kidney injury via decreasing mitochondrial ros and NLRP3 inflammasome activation	489
79	Vilaysane, A	2010	Journal of The American Society of Nephrology	The NLRP3 inflammasome promotes renal inflammation and contributes to CKD	478
80	Mishra, BB	2013	Nature Immunology	Nitric oxide controls the immunopathology of tuberculosis by inhibiting NLRP3 inflammasome-dependent processing of IL-1β	477
81	Gaidt, MM	2017	Cell	The DNA inflammasome in human myeloid cells is initiated by a sting-cell death program upstream of NLRP3	474
82	Wu, XX	2018	Cell Death & Disease	Nicotine promotes atherosclerosis via ROS-NLRP3-mediated endothelial cell pyroptosis	473
83	Hise, AG	2009	Cell Host & Microbe	An essential role for the NLRP3 inflammasome in host defense against the human fungal pathogen candida albicans	472
84	Coll, RC	2022	Trends in Pharmacological Sciences	NLRP3 and pyroptosis blockers for treating inflammatory diseases	462
85	Gurung, P	2014	Journal of Immunology	FADD and caspase-8 mediate priming and activation of the canonical and noncanonical NLRP3 inflammasomes	461
86	Shi, HX	2016	Nature Immunology	NLRP3 activation and mitosis are mutually exclusive events coordinated by NEK7, a new inflammasome component	460
87	Yao, CX	2018	Circulation	Enhanced cardiomyocyte NLRP3 inflammasome signaling promotes atrial fibrillation	457
88	Broz, P	2010	Journal of Experimental Medicine	Redundant roles for inflammasome receptors NLRP3 and NLRC4 in host defense against salmonella	457
89	Villani, AC	2009	Nature Genetics	Common variants in the NLRP3 region contribute to crohn’s disease susceptibility	448
90	Yazdi, AS	2010	Proceedings of The National Academy of Sciences of The United States of America	Nanoparticles activate the NLR pyrin domain containing 3 (NLRP3) inflammasome and cause pulmonary inflammation through release of IL-1α and IL-1β	444
91	Sandanger, O	2013	Cardiovascular Research	The NLRP3 inflammasome is up-regulated in cardiac fibroblasts and mediates myocardial ischaemiareperfusion injury	440
92	Li, N	2019	Redox Biology	STING-IRF3 contributes to lipopolysaccharide-induced cardiac dysfunction, inflammation, apoptosis and pyroptosis by activating NLRP3	438
93	Malireddi, RKS	2019	Frontiers in Cellular And Infection Microbiology	ZBP1 and TAK1: Master regulators of NLRP3 inflammasome/pyroptosis, apoptosis, and necroptosis (pan-optosis)	437
94	Dempsey, C	2017	Brain Behavior and Immunity	Inhibiting the NLRP3 inflammasome with MCC950 promotes non-phlogistic clearance of amyloid-β and cognitive function in APP/PS1 mice	435
95	Chen, ML	2017	Journal of The American Heart Association	Trimethylamine-n-oxide induces vascular inflammation by activating the NLRP3 inflammasome through the SIRT3-SOD2-MTROS signaling pathway	434
96	Heid, ME	2013	Journal of Immunology	Mitochondrial reactive oxygen species induces NLRP3-dependent lysosomal damage and inflammasome activation	434
97	Rühl, S	2015	European Journal of Immunology	Caspase-11 activates a canonical NLRP3 inflammasome by promoting k+ efflux	430
98	Marchetti, C	2018	Proceedings of The National Academy of Sciences of The United States of America	OLT1177, a ß-sulfonyl nitrile compound, safe in humans, inhibits the NLRP3 inflammasome and reverses the metabolic cost of inflammation	427
99	Biasizzo, M	2020	Frontiers in Immunology	Interplay between NLRP3 inflammasome and autophagy	422
100	Chen, IY	2019	Frontiers in Microbiology	Severe acute respiratory syndrome coronavirus viroporin 3a activates the NLRP3 inflammasome	421

The 100 most-cited articles involve 811 authors. As shown in **Table 2**, the top 10 authors with the highest number of published articles were Latz E (11 articles), Fitzgerald KA (10 articles), Nunez G (10 articles), Zhou RB (7 articles), Kanneganti TD (6 articles), Schroder K (6 articles), Tschopp J (5 articles), Coll RC (4 articles), Franchi L (4 articles), Hornung V (4 articles). The top 10 authors with the highest citation frequency were Latz E (15717), Nunez G (13160), Fitzgerald KA (11217), Zhou RB (8924), Tschopp J (7182), Stutz, A (6677), Schroder K (6572), Hornung V (6502), He Y (5614), Bauernfeind FG (5502). Meanwhile, we also counted the number of articles and the citation frequency of the first author. As shown in **Table 2**, the first author with the highest number of published articles was Coll RC (3 articles). The first author with the highest citation frequency was Zhou RB (4406).

**Table 2:** Top 10 authors, countries, journals, and institutes in the top 100 cited articles.

Rank	Author	Number of studies	Total citation	Average citation
1	Latz E	11	15717	1429
2	Fitzgerald KA	10	11217	1122
3	Nunez G	10	13160	1316
4	Zhou RB	7	8924	1275
5	Kanneganti TD	6	3698	616
6	Schroder K	6	6572	1095
7	Tschopp J	5	7182	1436
8	Coll RC	4	4349	1087
9	Franchi L	4	5182	1296
10	Hornung V	4	6502	1626
Rank	Country	Number of studies	Total citation	Average citation
1	USA	65	61362	944
2	Germany	18	20093	1116
3	China	18	11570	643
4	Australia	13	13806	1062
5	Switzerland	12	13131	1094
6	Japan	8	5288	661
7	France	6	4867	811
8	Belgium	5	3943	789
9	Canada	5	4277	855
10	South Korea	5	3549	710
Rank	Journal	Number of studies	Total citation	Average citation
1	Nature	10	15131	1513
2	Nature immunology	9	6908	768
3	Immunity	8	7637	955
4	Cell	6	3686	614
5	Journal of immunology	6	4851	809
6	Nature medicine	6	8515	1419
7	Proceedings of the national academy of sciences of the united states of america	4	2167	542
8	Cellular molecular immunology	3	2555	852
9	Frontiers in immunology	3	1475	492
10	Journal of experimental medicine	3	1710	570
Rank	Institute	Number of studies	Total citation	Average citation
1	University of massachusetts	16	19183	1199
2	University of lausanne	10	12169	1217
3	University of michigan	10	13160	1316
4	Harvard university	9	8829	981
5	University of bonn	9	13861	1540
6	St. jude children’s research hospital	7	5244	749
7	University of queensland	6	5055	843
8	University of science and technology of china	6	4114	686
9	University of north carolina	5	6883	1377
10	CNRS	4	3239	810

The 100 most-cited articles from 25 countries. As shown in **Table 2**, the top 10 countries with the highest number of published articles were USA (65 articles), Germany (18 articles), China (18 articles), Australia (13 articles), Switzerland (12 articles), Japan (8 articles), France (6 articles), Belgium (5 articles), Canada (5 articles), South Korea (5 articles). The top 10 countries with the highest citation frequency were the USA (61362), Germany (20093), Australia (13806), Switzerland (13131), China (11570), Japan (5288), Norway (4924), France (4867), Canada (4277), and Ireland (4149).

The 100 most-cited articles from 46 journals. As shown in **Table 2**, the top 10 journals with the highest number of published articles were Nature (10 articles), Nature Immunology (9 articles), Immunity (8 articles), Cell (6 articles), Journal of Immunology (6 articles), Nature Medicine(6 articles), Proceedings of The National Academy of Sciences of The United States of America (4 articles), Cellular Molecular Immunology (3 articles), Frontiers in Immunology (3 articles), Journal of Experimental Medicine (3 articles). The top 10 journals with the highest citation frequency were Nature (15131), Nature Medicine (8515), Immunity (7637), Nature Immunology (6908), Journal of Immunology (4851), Nature Reviews Immunology (4799), Cell (3686), Cellular Molecular Immunology(2555), International Journal of Molecular Sciences (2486), Proceedings of The National Academy of Sciences of The United States of America (2167).

The 100 most-cited articles from 224 institutions. As shown in **Table 2**, the top 10 institutions with the highest number of published articles were University of Massachusetts (16 articles), University of Lausanne (10 articles), University of Michigan (10 articles), Harvard University (9 articles), University of Bonn (9 articles), St. Jude children’s research hospital (7 articles), University of Queensland (6 articles), University of Science and Technology of China (6 articles), University of North Carolina (5 articles), and CNRS (4 articles). The top 10 institutions with the highest citation frequency were University of Massachusetts (19183), University of Bonn (13861), University of Michigan (13160), University of Lausanne (12169), Harvard University (8829), University of Munich (7022), University of North Carolina (6833), St. Jude children’s research hospital (5244), University of Queensland (5055), Norwegian University of Science and Technology (4924).

**Figure 3:**
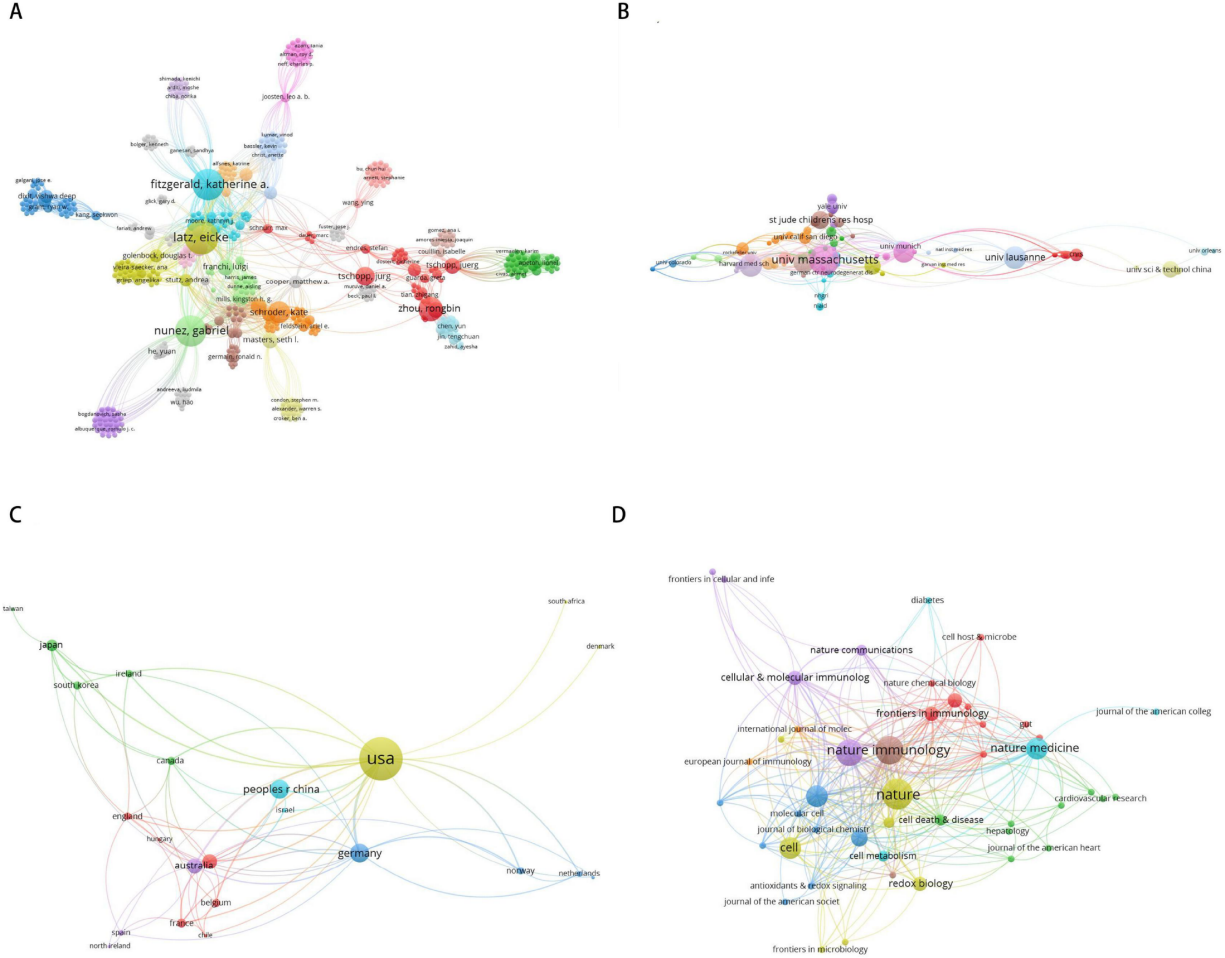
**(A)** Visualization of author network. **(B)** Visualization of institution network. **(C)** Visualization of country network. **(D)** Visualization of journal network.

**Figure 4:**
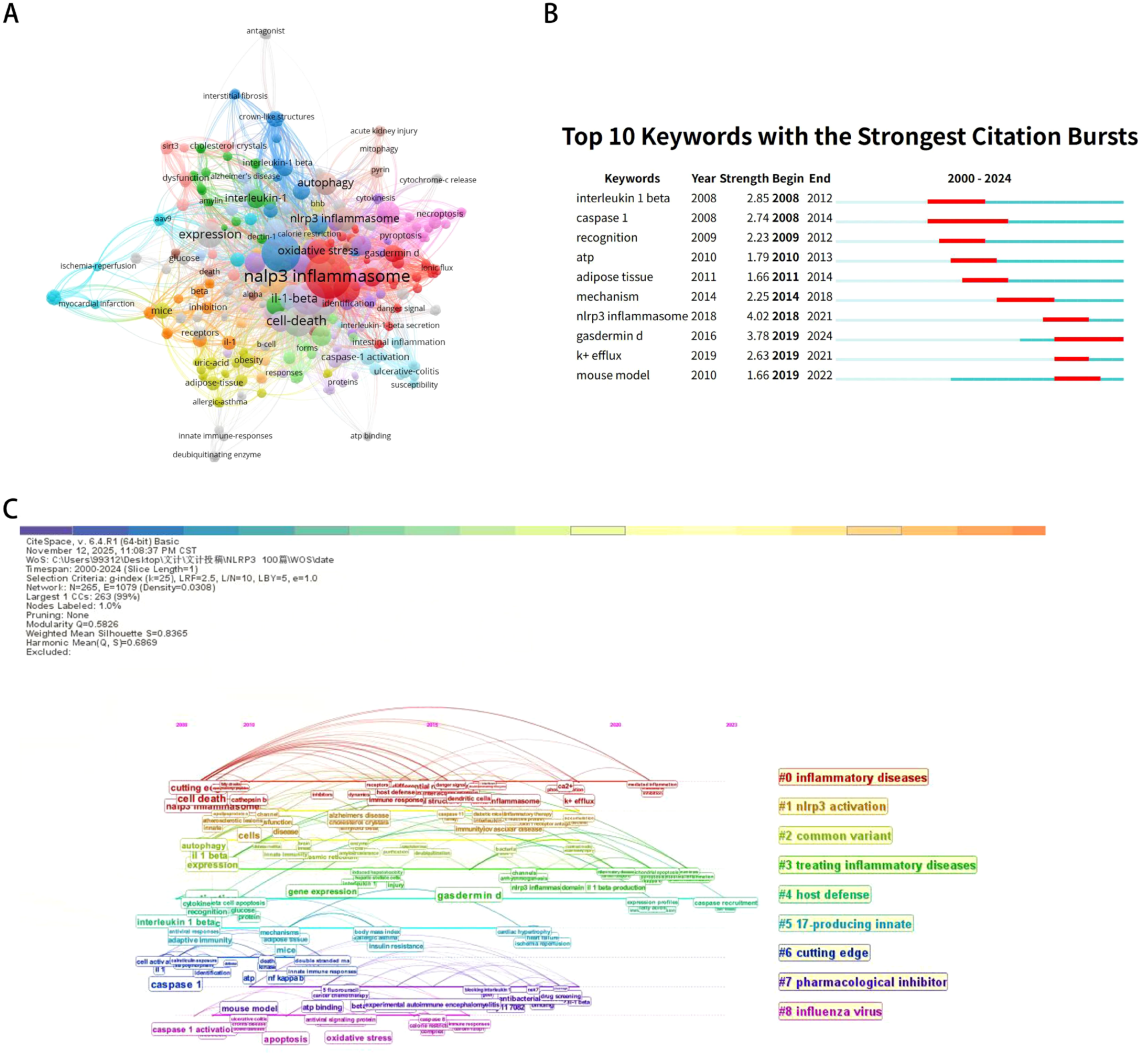
**(A)** Visualization of the keyword network. **(B)** Top 10 keywords with the strongest citation bursts. **(C)** Timeline view of keyword clusters.

To understand the collaborative networks among authors, institutions, countries, and journals in the field of NLRP3, co-authorship maps were generated using VOSviewer software. In the maps, circles and labels form an element. The size of the element depends on the degree of the node and the strength of the connection line. The color of the element represents the different clusters. Among these collaborative relationships, a larger node indicates a greater number of publications, and more connections suggest closer collaboration. As shown in **Figure 3A**, Latz E (151) had the strongest co-authorship ties. As shown in **Figure 3B**, University of Massachusetts (63) had the strongest co-authorship ties. As shown in **Figure 3C**, USA (64) had the strongest co-authorship ties. As shown in **Figure 3D**, Nature (153) had the strongest co-authorship ties. High-frequency keywords are words that appear frequently in the literature. Analyzing them helps identify research hotspots and trends within a field. Tracking changes in these keywords also aids in understanding the development of discipline. Our analysis identified 522 keywords. A co-occurrence analysis of high-frequency keywords was conducted using VOSviewer. Among these keywords, the size of a node corresponds to the frequency of the keyword, and the number of connections reflects its co-occurrence with other terms. As shown in **Figure 4A**, the top three high-frequency keywords were “NLRP3 inflammasome”, “activation”, and “Caspase-1”. According to total link strength, the top three keywords were the same. Emergent words refer to the keywords whose frequency of appearance increases significantly within a certain period. Analyzing these emergent words helps identify emerging trends and shifts in the research frontier. CiteSpace is a software for visualizing and analyzing trends in scientific literature, developed by Professor Chen Chaomei and his team. In this study, CiteSpace was used to conduct emergent words analysis and draw the emergent map. The blue line represents the timeline, while the red segments indicate the sudden duration of the keywords. The “Start” and “End” indicate the starting and ending years of the outbreak. As shown in **Figure 4B**, the period of keyword bursts spans from 2008 to 2022, with burst strength ranging from 1.66 to 4.02. Notably, “NLRP3 inflammasome” exhibit the highest burst strengths. Keyword burst analysis revealed shifting research priorities over time. The transition from “caspase-1” and “ASC” (early structural components) to “MCC950” and “NEK7” (therapeutic targets and regulatory mechanisms) illustrates the field’s maturation. Notably, “MCC950” appears exclusively in post-2015 publications, reflecting the shift toward translational research. A keyword timeline graph displays the distribution of keywords within clusters over time. We used CiteSpace to generate this graph. As shown in **Figure 4C**, the keywords were divided into 9 clusters. The results indicate a modularity value of 0.5826 (Q > 0.3) and a weighted mean silhouette score of 0.8365 (S > 0.5), suggesting a significant clustering structure and a reasonable clustering results.

## Mechanistic insights and discussion of the NLRP3 inflammasome from highly cited papers

**Figure 5:**
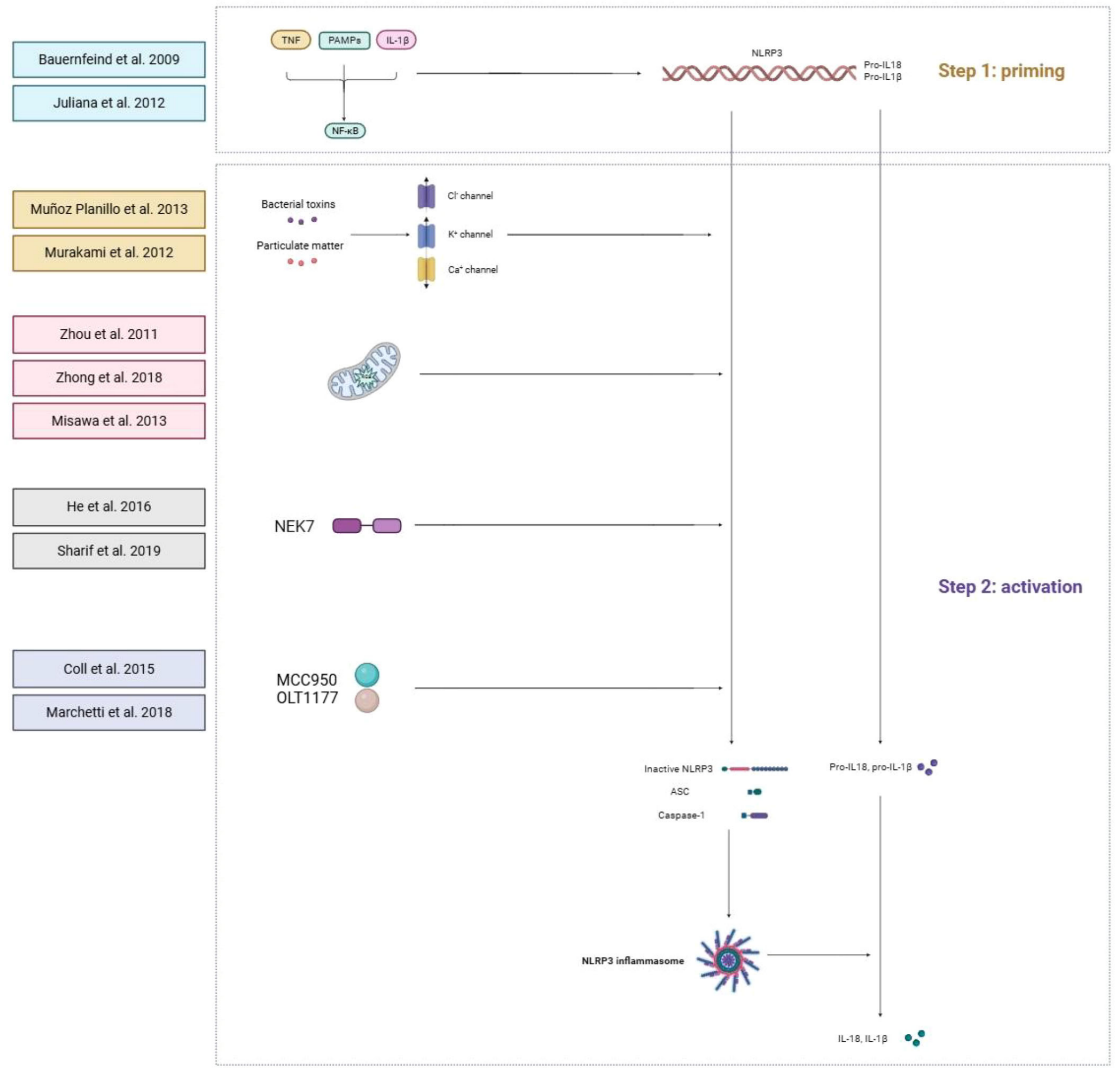
Mechanistic insights of NLRP3 from highly cited papers.

The top 100 most-cited articles not only reflect publication trends but also encapsulate the major mechanistic discoveries that have shaped our understanding of NLRP3 inflammasome biology. By analyzing these papers, we identified four key mechanistic themes that represent foundational knowledge in the field (**Figure 5**):

1. The two-step activation paradigm: The concept of priming and activation, established by Bauernfeind FG, remains a cornerstone of NLRP3 biology (20). This study demonstrated that NF-κB-mediated upregulation of NLRP3 expression is a prerequisite for inflammasome assembly, explaining how microbial products and cytokines license inflammasome responsiveness. Subsequent work by Juliana C revealed that non-transcriptional mechanisms, including deubiquitination of NLRP3 (21).
2. Ion fluxes as universal activation triggers:A unifying mechanism emerged from studies identifying potassium efflux as a common requirement for NLRP3 activation. Muñoz-Planillo et al. demonstrated that K^+^ efflux alone, induced by bacterial toxins or particulate matter, is sufficient for NLRP3 activation (22). This finding explained how structurally diverse stimuli converge on a single ionic event. Subsequent studies expanded this paradigm to calcium mobilization and chloride efflux, establishing ion homeostasis as a central regulatory node (23).
3. Organellar dynamics in inflammasome regulation: Mitochondria emerged as critical platforms for NLRP3 activation. Zhou et al. showed that mitochondrial reactive oxygen species (mtROS) and oxidized mitochondrial DNA released during mitochondrial dysfunction directly activate NLRP3 (24). This work positioned mitochondria as central integrators of cellular stress signals. Zhong et al. further revealed that new mitochondrial DNA synthesis, regulated by the transcription factor TFAM, is required for optimal NLRP3 activation, linking mitochondrial biogenesis to inflammasome responses (25). Additionally, Misawa et al. demonstrated that microtubule-mediated mitochondrial positioning facilitates NLRP3-ASC interactions, revealing cytoskeletal coordination of inflammasome assembly (26).
4. Structural basis of NLRP3 activation: Recent structural studies have provided atomic-level insights into NLRP3 regulation. The discovery of NEK7 revealed that cell cycle proteins regulate inflammasome activation (27). Cryo-EM structures of the NLRP3-NEK7 complex demonstrated that NEK7 binding stabilizes the active conformation of NLRP3, explaining the mutual exclusivity of mitosis and inflammasome activation (28). These structural insights have guided the development of allosteric inhibitors targeting protein-protein interactions.
5. The translational bridge: from mechanism to therapy: The characterization of specific NLRP3 inhibitors represents the culmination of mechanistic understanding. Coll et al. identified MCC950 as a selective NLRP3 inhibitor that binds the ATP-hydrolysis motif, preventing inflammasome assembly without affecting other inflammasomes (29). Similarly, Marchetti et al. demonstrated the clinical potential of OLT1177, a β-sulfonyl nitrile compound that safely inhibits NLRP3 in humans (30). These translational successes validate the therapeutic relevance of mechanistic discoveries.

In conclusion, our analysis reveals that citation patterns correlate with conceptual breakthroughs: papers introducing new mechanisms (ion fluxes, organellar signaling) or enabling tools (MCC950, NEK7) achieve sustained high citation rates. This observation supports the concept that citation metrics, despite their limitations, can serve as proxies for scientific impact when interpreted within a mechanistic framework.

## Analysis and discussion

As the most popular inflammasome, NLRP3 inflammasome has attracted significant attention in both scientific research and clinical fields in recent years (31, 32). By conducting a systematic analysis of the top 100 most-cited articles in this field, we can gain a deep understanding of its research hotspots, development trends, and academic collaboration networks. This study identified the top 100 most-cited articles in the NLRP3 field between 2000 and 2024. These articles covered 20 publishers, 218 funds, 19 research fields, 811 authors, 25 countries, 46 journals, and 224 institutions. References from 2024-2025 were included to capture the most recent developments in NLRP3 research. These articles were evaluated for their relevance to the historical context and were included only when they provided substantial reviews of the field’s evolution.

Among these authors, Latz E published the largest number of articles and had the highest citation frequency. This indicates that the author is not only a highly productive scholar in this field, but also that his research results have a high academic influence. Among these countries, the USA published the largest number of articles and had the highest citation frequency. The analysis at the national level reveals that the research on NLRP3 is still dominated by developed countries. Among these institutions, the University of Massachusetts published the largest number of articles and had the highest citation frequency. This institution usually has well-established research platforms, abundant clinical resources, and cross-disciplinary cooperation mechanisms, which provide support for its leading position in this field. The dominance of the USA, University of Massachusetts, and authors like Latz E in NLRP3 research reflects multiple interrelated factors. First, the USA’s leadership can be attributed to sustained funding from the National Institutes of Health (supporting 57 of the top 100 articles) and a well-established immunology research infrastructure. Second, the concentration at institutions like University of Massachusetts reflects the presence of pioneering researchers (Fitzgerald KA, Latz E) who established foundational concepts in inflammasome biology during the critical 2008-2012 period. This concentration of research output in high-income countries raises important questions about global equity in scientific contribution. While China appears in the top 3 countries by publication count (18 articles), its average citations per article (643) lag behind Germany (1116) and Switzerland (1094), suggesting that quantity does not always equate to impact. This disparity may reflect differences in research infrastructure, international collaboration networks, or publication strategies. Among these journals, Nature published the largest number of articles and had the highest citation frequency. This journal is the most influential scientific journal in the world. The predominance of English-language, high-impact journals (Nature, Immunity, Cell) in the top 100 list introduces potential bias. These journals preferentially publish groundbreaking mechanistic studies, potentially overlooking valuable clinical or translational research that might appear in specialized journals. Additionally, research from non-English speaking countries may be under-represented if published in local languages, as our search was limited to English-language publications. In addition, the top 100 most-cited articles mainly focus on Immunology, Biochemistry Molecular Biology, and Cell Biology, indicating that the research on NLRP3 has a strong interdisciplinary nature. The results of the collaboration analysis reveal a well-defined but potentially concentrated research landscape in the NLRP3 field. The co-authorship maps identify key central players, with specific individuals like Latz E, institutions like the University of Massachusetts, and countries like the USA serving as the most prolific and highly connected hubs. This indicates that the flow of knowledge and collaborative efforts are channeled through these central nodes, suggesting they are likely driving the research agenda. While this structure fosters strong, established networks, it might also highlight a reliance on a few key contributors. The journal network further underscores this focus, with high-impact journals like Nature being central platforms for dissemination.

By analyzing 100 highly cited papers, we have revealed the significant trend evolution in the field of NLRP3 research over the past nearly two decades. These trends can be divided into three stages, and our bibliometric data can also provide empirical support for them: The first stage (Main period: 2008-2012) is the era of mechanism elucidation. During this stage, research focused on answering the most fundamental questions: How is the NLRP3 inflammasome activated? Specific work included the discovery of key activation signals, the identification of various activators, and the formation of the “two-step activation” model. The success of this stage lies in constructing the core molecular framework for NLRP3 activation, laying the foundation for the explosive growth of the entire field (Zhou RB 2011, Bauernfeind FG 2009). The second stage (Main period: 2013-2018) is the era of pathophysiological expansion. During this stage, research shifted rapidly from immunological mechanisms (40% of all articles) to clarifying the core role of NLRP3 in major chronic human diseases. Specific work included exploring the key roles of NLRP3 in metabolic diseases, neurodegenerative diseases, cardiovascular diseases, autoinflammation, and autoimmune diseases (Heneka MT 2013, Mangan MSJ 2018). The success of this stage lies in positioning NLRP3 as a core inflammatory hub for multiple diseases, significantly enhancing its clinical translational value. The third stage (Main period: 2019-present) is the era of translational application and deep mechanism. The forefront and hotspots of this stage focus on developing NLRP3-specific inhibitors and promoting their clinical application. Currently, inhibitors such as MCC950 have been discovered and characterized (Coll RC 2019), and some inhibitors (DFV890) have entered the clinical stage (Yang Y 2019). Unfortunately, this stage faces obstacles in clinical translation, as well as the complexity of biological and regulatory networks.

This study also has some limitations: First, the literature included in this paper is limited to English journals. While English is the dominant language of scientific communication, important contributions published in Chinese, German, French, or other languages may have been omitted, potentially underrepresenting research from non-English speaking countries. Second, this study takes the Web of Science as the retrieval database. Although the information in the Web of Science database are the most comprehensive compared to other databases, important literature may still be omitted. Third, the publication time of an article does have an impact on its citation frequency. In recent years, some high-quality and high-standard articles may have been overlooked. Citation lag disproportionately affects recently published high-quality work. Articles from 2023-2024, despite potential exceptional quality, have had insufficient time to accumulate citations and may be underrepresented in our analysis. This “time bias” is inherent to citation-based bibliometric studies but should be considered when interpreting recent trends.

The current research on NLRP3 is characterized by the transformation from basic mechanism exploration to clinical practice. Future research should revolve around the development of NLRP3 inhibitors or NLRP3 activators. In addition, NLRP3 is not exclusively expressed in macrophages (33, 34). Future studies will focus more on its function in non-immune cells.

## Data Availability

The original contributions presented in the study are included in the article/supplementary material. Further inquiries can be directed to the corresponding authors.
